# Matrix-assisted autologous chondrocyte transplantation for treatment of focal chondral lesions in the knee: the *Hospital Israelita Albert Einstein* experience

**DOI:** 10.31744/einstein_journal/2022AO6819

**Published:** 2022-04-28

**Authors:** Alessandro Rozim Zorzi, Eliane Antonioli, Camila Cohen Kaleka, Moisés Cohen, Juliana Aparecida Preto de Godoy, Andrea Tiemi Kondo, José Mauro Kutner, Mario Lenza, Mario Ferretti

**Affiliations:** 1 Hospital Israelita Albert Einstein São Paulo SP Brazil Hospital Israelita Albert Einstein, São Paulo, SP, Brazil.

**Keywords:** Cartilage, Knee injuries, Pain, Chondrocytes, Cell- and tissue-based therapy, Regenerative medicine

## Abstract

**Objective:**

Phase 1 clinical trial to determine feasibility, safety, and efficacy of a new advanced cell therapy product for treatment of knee articular cartilage injuries.

**Methods:**

Three participants with knee focal chondral lesions were included, with no signs of osteoarthritis. Chondrocytes were obtained through knee arthroscopy, cultured in collagen membrane for 3 weeks at the laboratory, subjected to tests to release the cell therapy product, and implanted. All patients underwent a specific 3-month rehabilitation protocol, followed by assessments using functional and imaging scales. The main outcome was the incidence of severe adverse events.

**Results:**

Three participants were included and completed the 2-year follow-up. There was one severe adverse event, venous thrombosis of distal leg veins, which was no associated with therapy, was treated and left no sequelae. The clinical and radiological scales showed improvement in the three cases.

**Conclusion:**

The preliminary results, obtained with the described methodology, allow concluding that this product of advanced cell therapy is safe and feasible. ReBEC platform registration number: RBR-6fgy76

## INTRODUCTION

Injuries to the articular cartilage pose a therapeutic challenge due to their low healing potential. They can cause debilitating symptoms, such as pain, joint effusion, locking, and clicking. They can even progress to osteoarthritis, which is the most common cause of chronic pain in adults.^(1-3)^Cell therapy has been an integral part of the evolution of treatment of articular cartilage injuries with the goal of restoring damaged hyaline cartilage.

The first results of autologous chondrocyte implantation were published in 1994.^(4)^ Although the initial results were controversial,^(5)^ replacing the periosteal seal used to cover the lesion and retain the chondrocytes with a membrane made of collagen improved the clinical results, decreasing the incidence of complications.^(6)^The new technique became known by the acronym MACI, matrix-assisted chondrocyte implantation (MACI; Genzyme Biosurgery, Cambridge, Massachusetts, USA). The U.S. Food and Drug Administration (FDA) approved the commercial use of MACI in 2016, based on the results of a phase 3 multicenter study conducted in Europe, which demonstrated superiority of this technique over microfracture.^(7)^ Since then, the product has been approved in many other countries, and is considered economically viable and the therapy of choice for lesions larger than 2cm in diameter.^(8-12)^

Unfortunately, to date, there has been no product similar to MACI authorized for clinical use in Brazil. In compliance with the regulations of the National Health Surveillance Agency (ANVISA - *Agência Nacional de Vigilância Sanitária*), we conducted a validation study of the good production practices of the advanced cell therapy product at our organization as part of a clinical study, to demonstrate the feasibility and safety of the operational procedures.

## OBJECTIVE

Phase 1 clinical trial to determine feasibility, safety, and efficacy of a new advanced cell therapy product for treatment of knee articular cartilage injuries.

## METHODS

### Study design and participants

This is an open-label, experimental, single-arm clinical trial to evaluate the feasibility and safety of an advanced cell therapy product (phase 1 study). The study was conducted between February 2018 and June 2021.

The study was approved by the Ethics Committee of *Hospital Israelita Albert Einstein* (HIAE),# 2,346,079 (CAAE: 73911617.2.0000.0071). The study protocol was previously published^(12)^ and entered into the ReBEC platform: RBR-6fgy76 http://www.ensaiosclinicos.gov.br/rg/RBR-6fgy76/.

**Figure 1:**
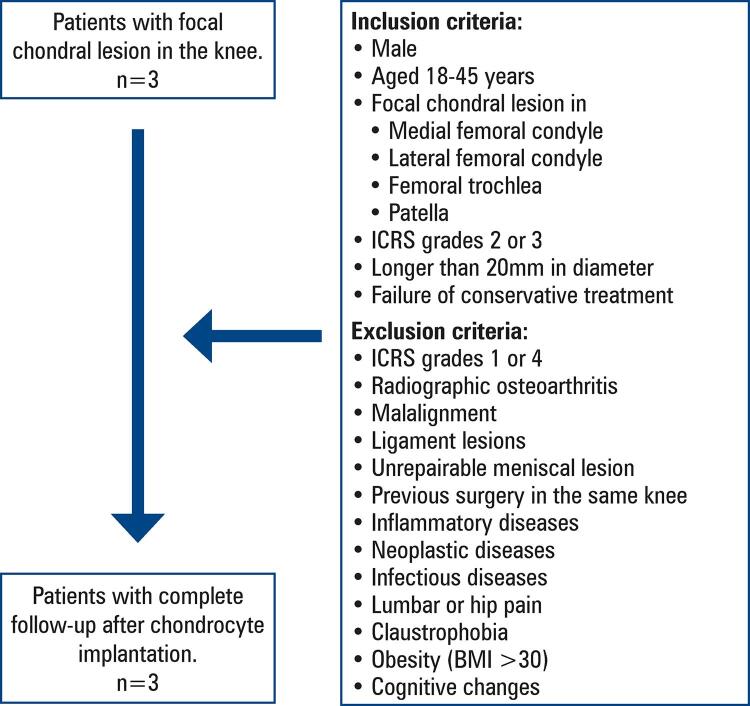
Inclusion and exclusion criteria of study participants

ICRS: International Cartilage Regeneration & Joint Preservation Society; BMI: body mass index.

Participant inclusion and exclusion criteria are shown in figure 1.

### Cartilage collection, isolation, and expansion of chondrocytes

**Figure 2:**
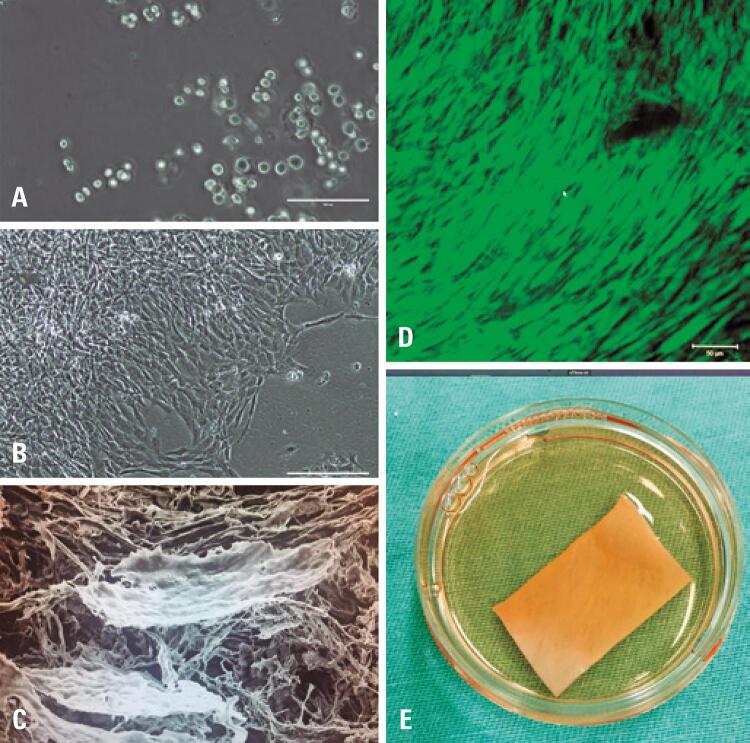
Images of the technique used in the laboratory for isolation and expansion of chondrocytes and preparation of the membrane. (A) Inverted light microscopy showing the low density of chondrocytes with spherical shape obtained soon after the digestion of the extracellular matrix; (B) Inverted light microscopy after expansion, with 80% confluence and star-shaped chondrocytes resembling the phenotype of fibroblasts (dedifferentiation); (C) Scanning electron microscopy showing the collagen fibers of the membrane and the presence of cells on its surface; (D) Confocal microscopy with Live/Dead® fluorescent marker, showing more than 90% cell viability in a fragment of membrane to be implanted in the patient; (E) Membrane with chondrocytes packed in Petri dish for transportation to the operating room

The steps of cartilage collection and chondrocyte isolation and expansion were performed as described in the protocol.^(13)^ Fragments of articular cartilage (100mg to 200mg) were collected from the intercondylar fossa by arthroscopy. Before the procedure, 200mL of blood were collected to obtain serum for chondrocyte culture. Chondrocytes were isolated from the cartilage fragments by digestion with collagenase type I enzyme. The chondrocytes were expanded in culture medium with 20% autologous serum until they reached the required number (1x10^6^cells/cm^2^) for adherence on collagen membrane types I and III (Chondro-Gide^®^, Geistlich Pharma). All production steps were performed in the cell therapy laboratory of HIAE, in ISO6-rated rooms, certified by ANVISA. Figure 2 illustrates the main steps involved in the preparation of the product, which range from isolation with digestion of the extracellular matrix and obtaining a low density of spherical chondrocytes, to expansion with an increase in the number of cells at the expense of the phenomenon of de-differentiation. After the expansion, the chondrocytes are seeded onto the membrane, and quality and safety tests are performed before the release and implantation of the product in the patient.

All samples were subjected to quality controls for the release of the advanced cell therapy product according to the technical standards in force, which included cell viability greater than 90%; unaltered karyotype analysis; negative culture for aerobic/anaerobic, fungii, and special microorganisms; negative result for mycoplasma; and endotoxin dosage less than 5EU/mL.

### Arthrotomy and implantation of membrane with cells

**Figure 3:**
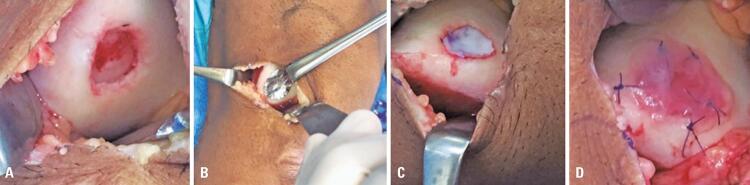
Images of the surgical technique used for autologous chondrocyte membrane implantation. (A) Focal chondral lesion immediately after debridement of the injured fragments, leaving vertical borders of healthy hyaline cartilage; (B) Metal mold for cutting the membrane in the exact shape and size of the lesion; (C) Placement of the membrane with chondrocytes over the lesion; (D) Fixation of the membrane with five stitches of absorbable 6-0 suture and fibrin glue

Surgery was performed according to a technique described and established in the literature.^(14)^ Briefly, access to the joint was performed by parapatellar arthrotomy without dislocation of the patella, medially or laterally, depending on the side of the lesion. The lesions were debrided to remove all abnormal tissue, taking care to preserve the calcified layer at the base to avoid bleeding, and obtaining vertical peripheral edges in healthy tissue. The membrane containing the chondrocytes was placed with the porous side facing the subchondral bone and sutured over the lesion with four to five stitches of 5-0 absorbable suture (Monocle^®^, Ethicon), and covered with fibrin glue (Tissucol, Baxter). The purpose of the fibrin glue is to hold the membrane in place, avoiding the need for a more laborious and time-consuming suture, as was done in the past. This allows a small arthrotomy with no need for patellar dislocation, leading to a more comfortable postoperative period for the patient. Fibrin glue is a biological material that is widely used in many surgical specialties. There is no report that its use may interfere with cartilage formation, and it is routinely used in countries where chondrocyte implantation is already used in clinical practice. The wound was closed in layers in the usual manner, and the incision was covered with dressing. No drains were used. Figure 3 illustrates the steps of the surgical procedure.

### Rehabilitation

In femoral condyle injuries, participants were instructed to walk with two crutches for 6 weeks. Immobilization was not used. The goal was to maintain full knee extension already in the first week and to achieve 90° flexion in 6 weeks. Between the 7^th^ and 12^th^ week after surgery, gradual weight-bearing progression was made until achieving full weight-bearing and complete range of motion. Between the 13^th^ and 16^th^ weeks, muscle strengthening, proprioception training, and functional activities were performed. The participant with trochlear lesions, kept immobilization orthosis in extension for 6 weeks, with physical therapy-assisted passive motion, starting at zero to 40°, with an increment of 5° per day. During this period, the participant used two crutches. After this, a protocol similar to the one used for condylar injuries was used. Full return to high-impact activities was only allowed one year after surgery, depending on the participant’s symptoms and clinical tests.

### Outcomes

Demographic data of the participants and injury characteristics were collected, such as age, sex, body mass index, injury site seen by magnetic resonance imaging, injury size, and concomitant injuries. A complete clinical examination of the gait and lower limbs was performed before surgery and monthly thereafter.

The safety of the procedure was assessed by the incidence of reoperations for any cause after chondrocyte membrane implantation, and by incidence and severity of complications according to version 4.0 of the National Cancer Institute (NCI) Common Terminology Criteria for Adverse Events (CTCAE) list.^(15)^

Efficacy was evaluated by means of clinical scales and imaging tests, as follows:

- Western Ontario and McMaster Universities Osteoarthritis Index (WOMAC) validated for Portuguese.^(16)^ The scale was answered by the participants, with no assistance from the researcher, before surgery, 6 months, 1 year, and 2 years after surgery.
- Subjective International Knee Documentation Committee (IKDC) clinical scale.^(17)^ The scale was answered by the participants, with no assistance from the researcher, at four timepoints: before, after 6 months, 1 year, and 2 years after surgery.
- Magnetic Resonance Observation of Cartilage Repair Tissue (MOCART) scale.^(18)^ Independent evaluations were performed by an orthopedic surgeon and a radiologist, with scans performed before and 12 months after surgery.

### Statistics

Since this is a phase 1 study to assess the feasibility and safety of the advanced cell therapy product, three participants were included, considering this is an advanced cell therapy product, with proven efficacy, already described in the literature, and with well-established use in clinical practice in countries in North America, Asia, and Europe. In a meeting held with ANVISA representatives, it was requested that this small number of cases be performed to demonstrate the laboratory’s local capacity to produce the chondrocyte implant.

Demographic data and outcomes were presented as mean and standard deviation for continuous variables and as absolute frequencies for categorical variables. All calculations were performed using the (SPSS) software, version 22.0 (IBM Corp., Armonk, NY, USA).

## RESULTS

### Participants

Three participants were included in the study. All underwent surgery and completed the 2-year follow-up period. Demographic data and details of the lesions, such as age, weight, laterality, time of evolution, location, and size of the lesion, depth and associated lesions, are described on table 1.

**Table 1:** Demographic data of the participants and description of the characteristics of the chondral lesions

Patient	Age (years)	Sex	BMI	Lesion	Side	Dimension* (mm)
I	30	Male	32	Lateral condyle	Left	19x13
II	44	Male	28	Trochlea	Right	10x6
III	36	Male	26	Trochlea	Left	15x13

* Size of the lesion on the preoperative the magnetic resonance imaging.

BMI: body mass index.

### Safety

Both arthroscopies for cartilage biopsy collection and arthrotomies for chondrocyte membrane implantation process went uneventfully.

The cell cultures were all completed within 3 weeks, never exceeding the second passage of the chondrocytes.

No case needed reoperation after implantation - one of the main outcomes of this study. There was no case of joint stiffness.

One participant had to have blood and cartilage collected twice. The day before implantation, the test for mycoplasma by reverse transcriptase polymerase chain reaction (RT-PCR) was positive. For safety reasons, the surgery was suspended, and the cell therapy product was discarded. Investigation of the source of contamination concluded the source was the autologous serum used for chondrocyte expansion. The participant had had an acute respiratory condition 2 weeks prior to blood draw and had not informed the researchers. The participant was followed by an infectious disease physician and treated with antibiotics. After testing negative for mycoplasma, the participant chose to continue in the study. Next, a new arthroscopy was performed to collect cartilage along with a new blood collection to obtain serum. After passing all the cell therapy product release tests, the participant received the implant. This adverse event was classified as mild.

Table 2 presents the type and frequency of adverse events. Only one severe adverse event was observed. The participant required further hospitalization after implantation because of a diagnosis of calf vein thrombosis on the sixth day after surgery. During the hospitalization period, the participant presented with asymptomatic pulmonary embolism detected on chest computed tomography scan. Followed by an angiologist and receiving treatment with anticoagulants for 6 months, the participant was discharged from vascular treatment with no sequelae. After investigating the occurrence of venous thrombosis, it was concluded that this event was unrelated to the use of the investigational cell therapy product. He was the only case that used immobilization after surgery due to the femoral trochlea lesion.

**Table 2:** List of adverse events that occurred during the study

Adverse event	Participant	Degree
New collection	II	Mild
Synovitis	III	Mild
DVT/PTE	III	Severe
Arthralgia	II	Mild
Rehospitalization	III	Severe
Reoperation	Zero	-
Joint stiffness	Zero	-

DVT: deep vein thrombosis; PTE: pulmonary thromboembolism.

### Efficacy

**Figure 4:**
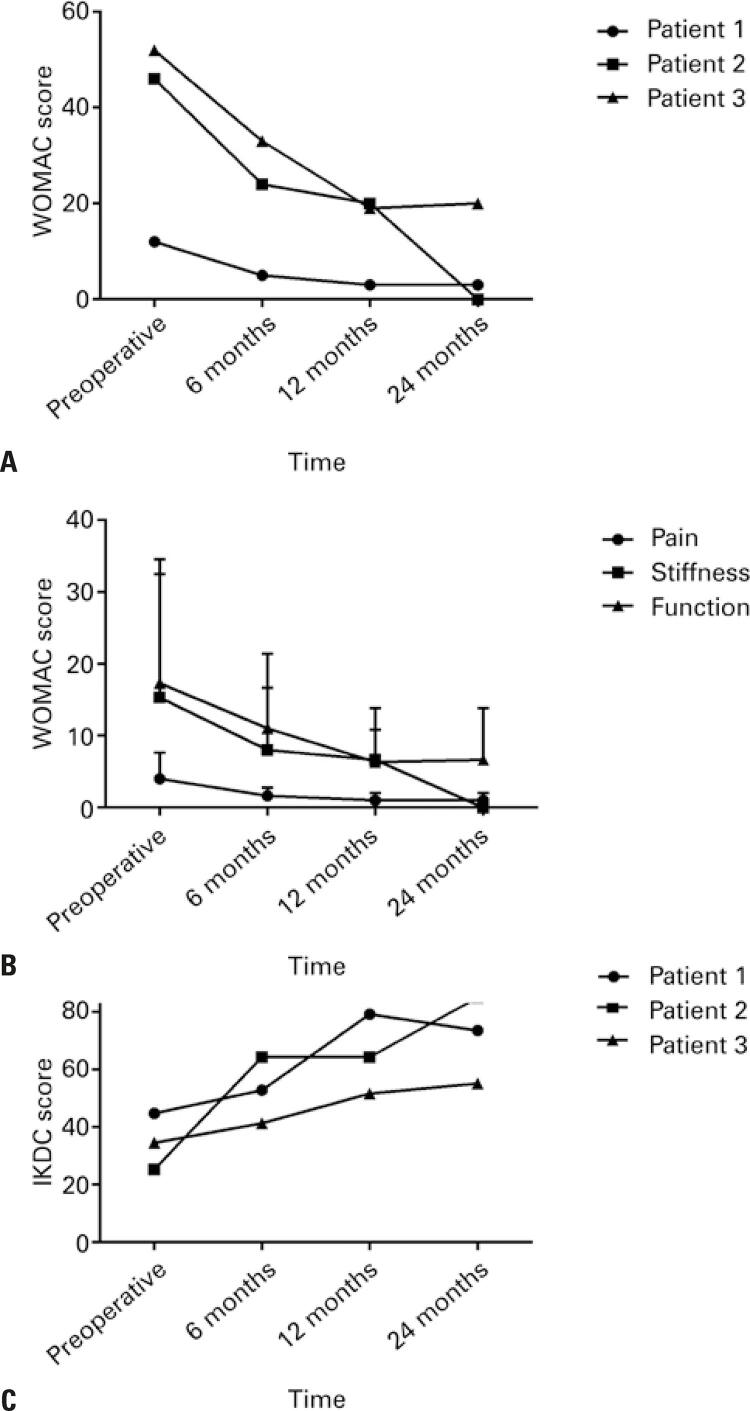
Bar charts with the results of the Western Ontario and McMaster Universities Osteoarthritis Index total (A), the Western Ontario and McMaster Universities Osteoarthritis Index sub-items (B), and the International Knee Documentation Committee scale (C) at four timepoints: preoperative and postoperative, 6, 12, and 24 months

WOMAC: Western Ontario and McMaster Universities Osteoarthritis Index; IKDC: International Knee Documentation Committee.

**Figure 5:**
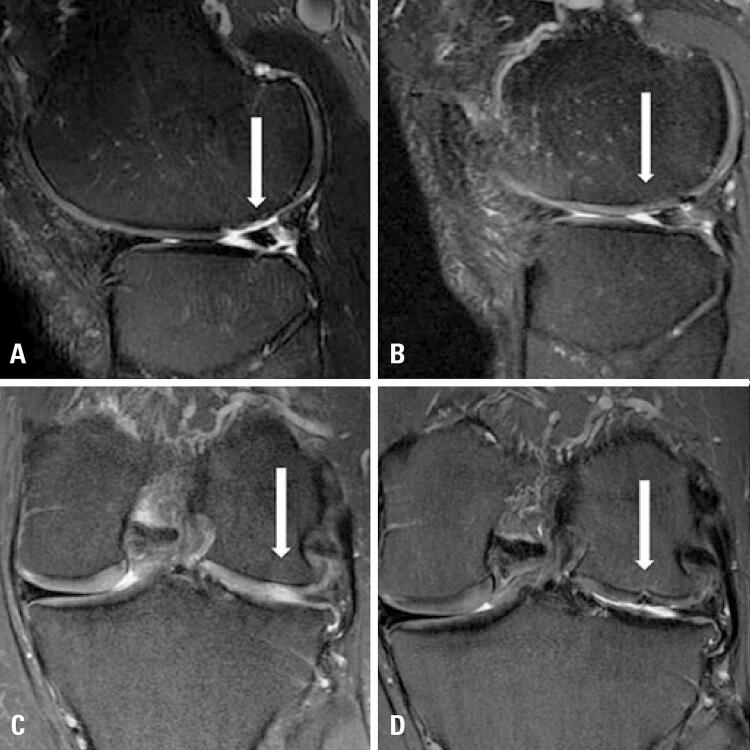
Magnetic resonance imaging of the lesions before and after 12 months of treatment. (A) Sagittal T2-weighted section before surgery, showing the focal chondral lesion (arrow); (B) Same section 12 months after chondrocyte implantation, demonstrating satisfactory filling of the lesion with tissue similar to hyaline cartilage around the lesion (arrow); (C) Same examination of figure 5A, but in frontal section; (D) Same examination of figure 5B, but in frontal section

Although not the primary objective of this study, assessments of the participants’ treatment efficacy were included. Figure 4 presents the results of the total WOMAC clinical indices (4A), WOMAC divided by subscales (4B), and the IKDC ladder (C). Figure 5 shows magnetic resonance images of the three cases before and after surgery. Table 3 presents the results of the MOCART scale. There was clinical and functional improvement in all three participants on all these scales.

**Table 3:** Results of evaluations by Magnetic Resonance Observation of Cartilage Repair Tissue

Patient	I	I	I	I	II	II	II	II	III	III	III	III
Evaluator	1	2	1	2	1	2	1	2	1	2	1	2
MOCART Timepoint	Pre-	Pre-	Post-	Post-	Pre-	Pre-	Post-	Post-	Pre-	Pre-	Post-	Post-
Filling	0	0	20	20	5	10	10	20	5	5	15	20
Cartilage	0	0	15	10	0	5	15	15	0	0	5	5
Surface	0	0	10	10	5	5	10	10	5	0	5	5
Adherence	5	5	5	5	5	5	5	5	5	5	5	5
Structure	0	0	5	5	0	0	5	0	0	0	0	0
Sign	0	0	10	10	0	0	10	10	0	0	10	10
Baseline	5	5	5	5	5	5	5	5	5	5	5	5
Subchondral Bone	5	0	5	0	0	0	5	0	5	5	5	0
Effusion	5	0	5	5	0	0	5	5	0	0	5	5
Total	20	10	80	70	20	30	70	70	25	20	55	55

MOCART: Magnetic Resonance Observation of Cartilage Repair Tissue.

## DISCUSSION

The main result of this study was to determine the safety and feasibility of the autologous chondrocyte implant produced according to the Brazilian regulatory standards for advanced therapy products. This was an important and necessary initial step to obtain product registration and to enable the clinical use of chondrocytes in our country.

According to the largest clinical study published so far,^(7)^ the most frequent complications of autologous chondrocyte membrane implantation are treatment failure, arthralgia, and joint effusion; the incidence of severe adverse event reported was 15.3%. In this study, there was only one case of joint effusion, but all three participants had successful treatments after one-year follow-up. We had one case of a severe adverse event - venous thromboembolism, which required hospitalization, but had no sequelae. After this case, the postoperative protocol was modified, introducing pharmacological venous thromboembolism prophylaxis.

Also regarding safety of chondrocyte implantation, a problem has been the heterogeneity of protocols, which leads to discrepant results according to the method of cell production, the surgical technique, and the rehabilitation protocol. A 5-year follow-up randomized clinical trial with 33 participants showed only two cases of treatment failure. In this study, two rehabilitation protocols were tested, one traditional and the other accelerated, with no difference in results.^(19)^ Another clinical trial, with the longest follow-up to date, accompanied participants for up to 11 years and evaluated the incidence of adverse events, showing an incidence of reoperations in 33.3% of participants, a very high rate.^(20)^ Another study analyzed a database with 315 patients operated for chondrocyte implantation and a mean follow-up time of 39.2 months, reporting an incidence of only 1.6% of complications.^(21)^

In Brazil, other studies on chondrocyte implantation have been published. In 2003, Lombello et al.^(22)^ described the results of a technique for isolation and culture of chondrocytes, but there was not yet a national regulatory standard. In 2008, the same group published the results of application of the technique in six patients, with good results.^(23)^Only one patient presented with joint stiffness and had to be reoperated to remove adhesions. In 2010, another group published the results of three cases. Although they did not describe the complications that occurred, they reported little improvement and maintenance of pain in all three cases, suggesting treatment failure.^(24)^ In 2020, Giglio et al.^(25)^ presented the results of 11 patients treated with chondrocytes in the membrane, with a different cell therapy product production protocol from this study. The authors reported the occurrence of complications in four cases: two evolved with adhesions and joint stiffness, requiring reoperations to recover motion; one had an infection of the surgical wound at the site of bone graft removal, and one presented with implant hypertrophy. It is believed that the high complication rate can be explained by inclusion of participants with broader criteria, such as those with other associated injuries.

The main advantages of this study were the definition of strict inclusion criteria, the emphasis on adherence of participants to a standardized rehabilitation protocol, and the design and execution of the study in accordance with ANVISA regulations, which allowed the production of an advanced therapy product comparable to those already marketed in other countries. Moreover, the 2-year follow-up is important because it is the time considered necessary for the graft to reach the expected maturity.^(26)^ The main limitations of this study were the small sample size and the lack of a control group, which do not allow conclusions as to the efficacy of the method. A protocol for a new phase 2 study with a larger sample size and control group is already being prepared.

## CONCLUSION

The results obtained in this initial study indicate safety and feasibility of the production method of autologous implantation of chondrocytes with membrane that is being developed at *Hospital Israelita Albert Einstein*. This result allows larger studies to demonstrate the efficacy of the technique. These steps are necessary for the autologous chondrocyte implant to be offered in clinical practice.
